# Editorial: Exploring effects of group and partnership dynamics in dance on mental and physical health

**DOI:** 10.3389/fpsyg.2025.1697858

**Published:** 2025-10-13

**Authors:** Deborah A. Jehu, Judith Bek, Madeleine E. Hackney

**Affiliations:** ^1^Department of Community & Behavioral Health Sciences, Institute of Public and Preventive Health, School of Public Health, Augusta University, Augusta, GA, United States; ^2^School of Psychology, University College Dublin, Dublin, Ireland; ^3^Centre for Motor Control, Faculty of Kinesiology & Physical Education, University of Toronto, Toronto, ON, Canada; ^4^Division of Geriatrics and Gerontology, Department of Rehabilitation, Emory University School of Medicine Department of Medicine, Atlanta, GA, United States; ^5^Atlanta VA Center for Visual and Neurocognitive Rehabilitation, Decatur, GA, United States; ^6^Birmingham/Atlanta VA Geriatric Research Education and Clinical Center, Atlanta, GA, United States

**Keywords:** dance, exercise, cognition, neuroplasticity, intervention, partnered dynamics, remote, digital

## Introduction

Dance is a universal human expression that transcends cultural and temporal boundaries. As an activity that engages motor, cognitive, neurophysiological, and psychological dimensions, dance has emerged as a rich field for interdisciplinary research. This Research Topic, comprising 14 diverse and insightful articles, explores the intricate interplay between dance and cognitive functioning, mental wellbeing, and physical health across varied populations and contexts. By delving into the mechanisms that underlie dance's transformative potential, these studies illuminate its role as both an artistic pursuit and a therapeutic tool, offering evidence-based insights into its capacity to enhance human health and resilience.

## Cognitive and neurophysiological dimensions of dance

Dance is a cognitively demanding activity that engages processes such as memory, attention, spatial awareness, and executive functioning. Neuroimaging research has identified key neural markers and adaptive brain changes associated with dance training, pointing to dance's role in fostering neuroplasticity and network connectivity (Hackney et al., 2024). Several articles in this Research Topic highlight the cognitive benefits of dance and the neurophysiological mechanisms that underpin them. Mao et al.'s ethnological analysis of the Tujia dance provides a compelling example, identifying nine therapeutic principles, including neuroplasticity and network connectivity, that underscore dance's capacity to reshape brain function. These findings align with the growing body of evidence suggesting that dance training enhances cognitive reserve, potentially mitigating age-related cognitive decline (Mitterová et al., 2021).

Yu et al.'s randomized controlled trial (RCT) with 90 female university students further illustrates dance's cognitive and physical benefits. Their study demonstrates that various dance genres improve strength, power, agility, and flexibility, with genre-specific benefits offering a rationale for integrating dance into higher education curricula. These findings suggest that dance not only bolsters physical fitness but also enhances cognitive processes critical for academic and professional success, such as attention and executive functioning.

Shimizu et al.'s experimental study of expert break dancers provides a novel perspective on the cognitive demands of dance in competitive contexts. By simulating a battle scene, Shimizu reveals how dancers coordinate movements and manage relative distances in a time- and context-dependent manner. This study highlights the unique cognitive and motor synchronization required in performing arts, which may distinguish dance from other interpersonal activities like sports. Such insights underscore the need for further research into the cognitive mechanisms that enable dancers to navigate complex, dynamic environments.

## Psychological and emotional benefits of dance

Dance can also have profound psychological benefits, influencing mood regulation, stress reduction, and mental resilience. Liu et al.'s systematic review of 16 articles demonstrates that dance interventions enhance physical self-esteem and self-confidence while reducing social physique anxiety among adults. Similarly, Li et al.'s study on Chinese children cared for by family members reveals that a 12-week dance intervention significantly improves social anxiety and self-concept, emphasizing dance's therapeutic potential in vulnerable populations. These findings suggest that dance fosters a positive self-image in an enjoyable, non-judgmental context, making it a powerful tool for addressing body image concerns and social anxiety.

Dwarika and Haraldsen's scoping review of 115 articles on mental health in dance provides a comprehensive overview of the field, identifying stressors, mental processes, and outcomes that conceptualize mental health as a dynamic state. Most of the articles focused on pre-professional ballet dancers, highlighting a gap in research on professional dancers across diverse styles, and calling for broader investigations to capture the complexity of mental health in dance.

Kawase and Eguchi's research on community festival dancing further underscores the psychological benefits of dance in fostering social bonding and reducing loneliness. Their findings indicate that dance-based festivals create stronger community ties than non-dance festivals, regardless of participants' prior festival attendance or willingness to participate. This highlights dance's unique ability to forge social connections, which are critical for mental wellbeing.

## Therapeutic applications of dance

The therapeutic potential of dance is a central theme of this Research Topic, with several articles exploring its application in clinical and community settings. Jehu et al.'s exploratory analysis of a randomized controlled trial examining tango vs. walking interventions for people with Parkinson's disease revealed that baseline social support influences physical function outcomes, with those having low social support benefiting more from walking interventions. This finding suggests that social context plays a critical role in the efficacy of dance-based interventions, warranting further exploration.

Bek et al.'s perspective piece on digital dance programs for Parkinson's disease highlights the feasibility and flexibility of virtual interventions, while acknowledging barriers such as limited internet access and reduced opportunities for creative expression, as well as important safety considerations. Furthermore, Qiu et al.'s study on digital dance therapy demonstrates its efficacy in reducing anxiety and enhancing positive emotions among both professional and amateur dancers, with widespread acceptance of digital tools. These findings suggest that digital platforms can expand access to dance therapy, though careful consideration of social and creative elements is needed to maximize benefits.

Jehu et al.'s perspective on group and partnered dance for people with dementia offers practical guidance on intervention design, emphasizing mirroring techniques, simple instructions, and caregiver support to enhance adherence and outcomes. Similarly, Fontanesi and Newman-Bluestein's opinion article advocates for movement therapy to support caregivers and individuals with dementia, highlighting its role in fostering group support, synchrony, and meaningful relational dynamics. These studies collectively underscore dance's potential to address neurological and psychological challenges, and to promote a shared sense of presence and connection.

## Interpersonal coordination and social dynamics in dance

The synergy between individuals in group and partnered dance provides a unique lens through which to explore social interaction, communication, and physical movement. Whitehead et al.'s study on interpersonal coordination in dance improvisation among healthy young dyads employs advanced methodologies, such as maximum correlation vectors and normalized symbolic transfer entropy, to measure movement dynamics and perception. Their work lays a foundation for future research using advanced quantitative methods into the complex interplay of motor and cognitive processes in dance.

Liu et al. address another critical gap by developing and validating the Partnership Scale-DanceSport Couples (PS-DSC) questionnaire, enabling researchers to quantify the dynamics of dance partnerships. This tool has significant implications for understanding how interpersonal relationships influence dance performance and psychological outcomes.

## Future directions and interdisciplinary synergy

**Figure 1:**
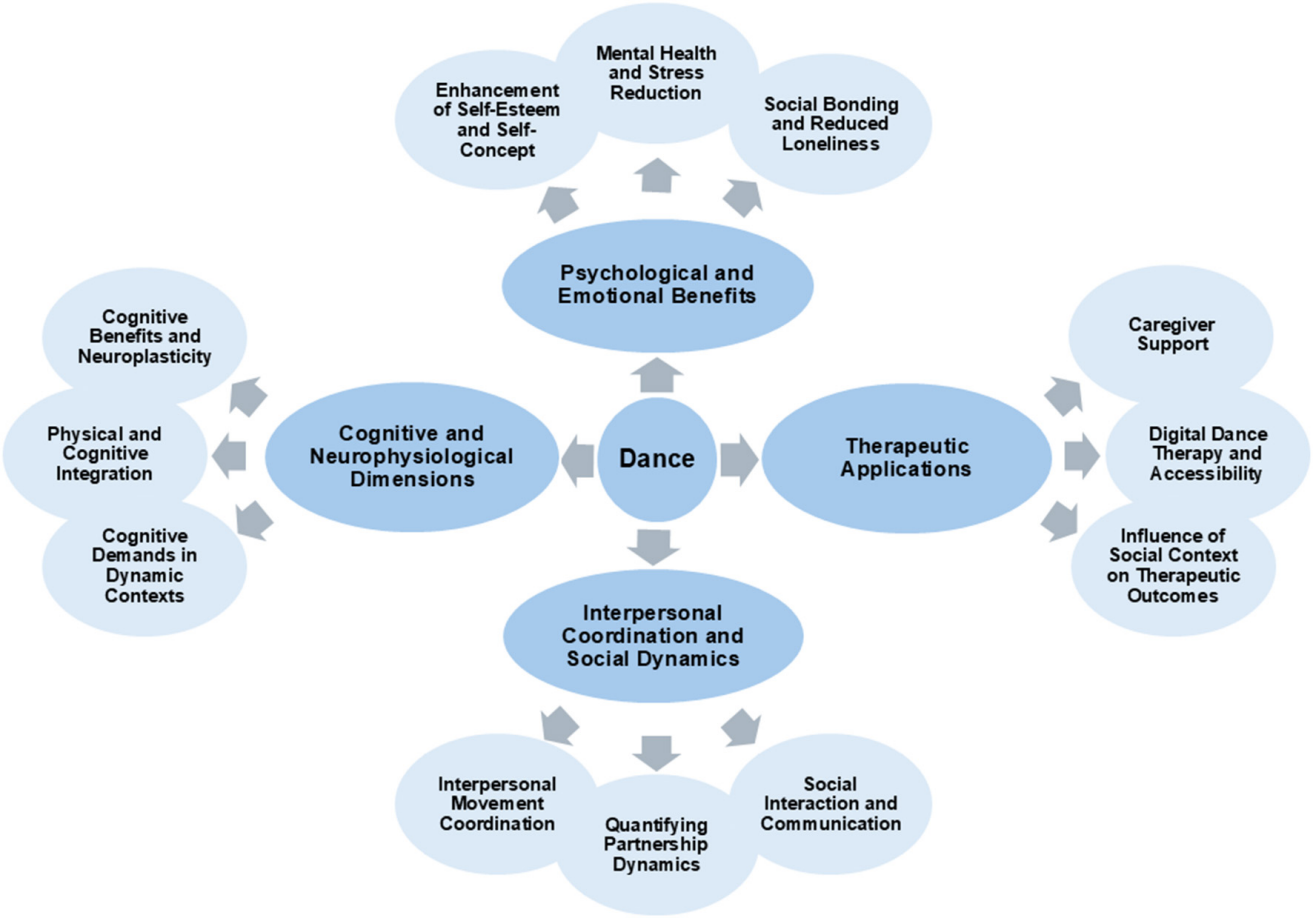
Conceptual diagram of topics exploring the psychological and emotional benefits, therapeutic applications, interpersonal coordination and social dynamics, and cognitive and neurophysiological dimensions of dance.

Figure 1 provides a conceptual diagram illustrating the multidimensional themes of dance research in this Research Topic. The literature in this Research Topic underscores the need for deeper interdisciplinary exploration to elucidate the mechanistic relationships linking dance to cognitive, psychological, and physiological outcomes. The diverse methodologies employed, including systematic reviews, RCTs, and ethnographic analyses, highlight the richness of this field. However, gaps remain, particularly in understanding the long-term effects of dance interventions, the role of specific dance genres, neural underpinnings of any effects, and the applicability of findings across diverse populations and cultural contexts.

The integration of cognitive neuroscience, neurophysiology, psychology, and movement science offers a promising path forward. For instance, combining neuroimaging with other biomarker analyses could further clarify the neural mechanisms underlying dance-induced brain adaptations. Similarly, longitudinal studies could elucidate the sustained impact of dance on mental health and cognitive function, particularly in clinical populations such as those with Parkinson's disease or dementia.

The therapeutic potential of dance warrants further exploration in both traditional and digital formats. As digital interventions become more prevalent, researchers must address barriers to access and ensure that these programs preserve the social and creative elements that make dance uniquely impactful. Finally, the development of standardized tools, such as the PS-DSC, will enable more rigorous investigations into the social dynamics of dance, fostering a deeper understanding of its role in promoting community and connection.

## Conclusion

This Research Topic represents a significant step toward unraveling the multifaceted impact of dance on human cognitive, psychological, and physical health. By bringing together cutting-edge research, theoretical perspectives, and evidence-based practices, these 14 articles illuminate the health benefits of dance across diverse populations. From enhancing self-esteem and reducing anxiety to fostering social bonding and promoting neuroplastic changes, dance emerges as a powerful tool for promoting mental and physical health. As we move forward, interdisciplinary collaboration and innovative methodologies will be essential to fully harness dance's potential as a transformative force in both artistic and therapeutic contexts. This Research Topic not only advances our understanding of dance but also inspires future research to explore the boundless possibilities in dance and health.
